# Procalcitonin: A Key Marker in Children with Urinary Tract Infection

**DOI:** 10.1155/2011/397618

**Published:** 2011-01-17

**Authors:** Sandrine Leroy, Alain Gervaix

**Affiliations:** ^1^Centre for Statistics in Medicine, Wolfson College Annexe, University of Oxford, Linton Road, Oxford OX2 6UD, UK; ^2^Pasteur Institute, 75015 Paris, France; ^3^Department of Pediatrics, University Hospital of Geneva, 1211 Geneva, Switzerland

## Abstract

Urinary tract infections (UTIs) are the most common source of bacterial infections among young febrile children. Accurate diagnosis of acute pyelonephritis (APN) and vesicoureteral reflux (VUR) is important because of their association with renal scarring, leading in the cases to long-term complications. However, the gold standard examinations for both are either DMSA scan (for APN and scar) or cystography (for VUR) and present limitations (feasibility, pain, cost, etc.). Procalcitonin, a reliable marker of bacterial infections, was demonstrated to be a good predictor of both renal parenchymal involvement in the acute phase and late renal scars. Furthermore, it was also found to be associated with high-grade VUR and was the key tool of a clinical decision rule to predict high-grade VUR in children with a first UTI. Therefore, procalcitonin may certainly be found playing a role in the complex and still debated picture of which examination should be performed after UTI in children.

## 1. Introduction

Urinary tract infections (UTIs) are the most common source of bacterial infections among young febrile children [1]; 7% of girls and 2% of boys will have a UTI before 6 years of age [2]. The nonspecific nature of symptoms among febrile infants and young children renders the clinical differentiation of upper and lower UTI difficult. However, accurate diagnosis and early treatment of acute pyelonephritis are important because of its association with renal scarring [3]. Thus, the belief that future complications related to renal scarring such as hypertension, pre-eclampsia, poor renal growth, and end-stage renal failure can occur have been a major driving force in the impetus to first make a prompt and high-quality diagnosis of UTI and then investigate the first occurrence of UTI for vesicoureteral reflux (VUR) and other urinary tract abnormalities [4, 5]. But which examination should be performed and in which order is a strongly debated topic for both early UTI diagnosis examinations and later investigations [6–8]. Among those, procalcitonin (PCT) should find a place in the diagnostic strategy for UTI. PCT, a 116-amino acid propeptide of calcitonin without hormonal activity, is a reliable marker of bacterial infections [9, 10]. In this paper, we aimed to review the clinical interest of PCT, and its place in the complex picture of which examination to prescribe in children with UTI.

## 2. Procalcitonin

PCT is the prehormone of calcitonin, which is normally secreted by the C cells of the thyroid in response to hypercalcemia. Under normal conditions, negligible serum PCT concentrations are detected [11], but PCT serum level rises during bacterial infections, as first demonstrated by Assicot et al. [9]. The mechanism proposed for PCT production after inflammation and its role are still not yet completely elucidated. It is believed that PCT is produced by the liver and peripheral blood mononuclear cells, modulated by lipopolysaccharides and sepsis-related cytokines. Microbial infections induce a ubiquitous increase in *CALC1* gene expression and a subsequent release of calcitonin precursors [12]. In bacterial infections, PCT increases from concentrations in the picogram range (below the detection level of current PCT assays) to plasma concentrations ranging from 1 to 1000 ng/mL. This increase often correlates with the severity of diseases and with mortality [9]. Moreover, PCT is very specific of bacterial infection and helps to distinguish between viral and bacterial infections which is particularly useful in children [10]. Increase in PCT occurs more rapidly than increase in CRP. PCT can be detected in the plasma 2 hours after the injection of endotoxins. Within 6–8 hours, PCT concentrations rise and a plateau is reached after approximately 12 hours. PCT decreases to its normal values after 2-3 days. This rapid and specific induction of PCT after an adequate stimulus, and the high and reliable production of PCT in patients with bacterial infections or sepsis, suggests a pathophysiological function of PCT in the acute immune response, even though it is not clear whether PCT is a cytokine, a hormone, or an acute-phase protein since it has characteristics of all these mediators.

PCT can be measured with a quantitative immunoluminometric assay (LUMItest PCT, progressively replaced by PCT sensitive KRYPTOR, both from Brahms Diagnostica, Berlin, Germany) in 2 hours, with a maximum interassay variation of approximately 0.3 ng/mL. A rapid semiquantitative chromatographic test (Brahms PCT-Q, Brahms Diagnostica) can be used at the bedside and gives an indication of PCT concentration (in bands of <0.5 ng/mL, 0.5–2 ng/mL, 2–10 ng/mL, and >10 ng/mL) in 30 min.

PCT has been used for one and a half decades in a number of hospitals as a clinical routine parameter for the diagnosis of sepsis [13] since its first demonstration by Assicot et al. [9]. There are several reasons for the clinical success of PCT. Plasma concentrations respond rather specifically to bacterial infection, and PCT has been demonstrated to be a better or at least of equal value for diagnosis of sepsis when compared with markers like CPR, lactate, proinflammatory cytokines, leukocytosis, and fever [10]. Not only diagnosis of sepsis but also diagnosis of specific infection can be improved by measuring PCT, as demonstrated by the meta-analysis comparing PCT and CRP in patients with bacterial versus viral or nonbacterial infections [10]. Moreover, PCT is helpful in monitoring the activity for the systemic inflammatory response, the success of therapy, and in estimating prognosis [10, 13].

## 3. PCT and Acute Pyelonephritis

The nonspecific nature of symptoms of acute pyelonephritis (APN) among febrile children renders the diagnosis of upper UTI difficult, whereas an accurate, quick, and readily available diagnostic test for an early and high-quality diagnosis of APN is of value. Renal scintigraphy with Tc-99m dimercaptosuccinic acid (DMSA) is considered as the gold standard for the demonstration of renal parenchymal involvement in children with a UTI [14], once the diagnosis of bacterial UTI remains indisputably based on the result of urine culture [4, 5]. However, not only does this modality entails irradiation, requires special equipment and facilities for its completion that are not easily available in a number of hospitals, but it is also expensive. Hence, a biological marker that would assist the clinician into predicting renal parenchymal involvement in a child with UTI would be valuable. Several teams conducted cohort studies to investigate the diagnostic accuracy of PCT for APN (Table 1) [15–27]. Most of authors concluded that PCT has a good diagnostic accuracy and an interesting clinical value for APN, with a sensitivity and a specificity ranging from 70 to 100% and 70 to 97%, respectively, across studies and thresholds [15–19, 21–27]. However, the Belgium team found lower sensitivity and specificity (68% and 23%, resp.) with no obvious difference regarding the cutoff or the population's characteristics [20]. Mantadakis et al. summarised most of these studies in a systematic review and meta-analysis (10 studies, *n* = 627) and demonstrated that the pooled diagnostic odds ratio (OR) measuring the association between DMSA-proven APN and PCT was 14.3 (95% confidence interval—CI, 4.7–42.2), after having pooled results from studies using close PCT threshold (0.5 and 0.6 ng/mL) [28]. However, this pooled result may be questionable regarding the strong heterogeneity it comes with (*Q*-test: *P* < .001; *I* ^2^ = 80%). Interestingly, an updated meta-analysis validated these results using individual patients data (17 studies, 13 centres, *n* = 1011); the relationship between APN and PCT ≥0.5 ng/mL after adjustment on all cofactors of interest was significant (adjusted OR = 6.4; 95% CI, 4.6–8.8), with 74% (95% CI, 71–78) sensitivity and 71% (95% CI, 66–75) specificity [29]. This is encouraging in building algorithms to identify children at high risk of acute pyelonephritis (i.e., with proven renal parenchymal involvement) once the diagnosis of UTI is made by urine culture in order to limit early DMSA scan to them. However, pieces of evidence and research are still needed before clinicians can use PCT as a daily and perfectly defined tool.

## 4. PCT and Renal Scarring

Beyond making a proper, high-quality, and prompt diagnosis of APN, the prediction of late renal scars is also of interest, as it may be related to future complications, such as hypertension, pre-eclampsia, poor renal growth, and end-stage renal failure [30]. Pediatricians aim to early identifying children with renal scars after UTI in order to propose a more specific followup to prevent the impairment of the renal function. The gold standard examination for scar is DMSA scan 6 to 12 months after UTI, and the same problems of irradiation, availability, and cost make it not so often performed. The PCT serum level at the time of UTI was then studied by several authors as a potential predictor for late renal scars (Table 2) [16–18, 20, 22, 26, 27, 31]. Zaffanello et al. reviewed these results in a nonsystematic review and concluded to conflicting findings [32]. However, a meta-analysis of the individual patient data (*n* = 361) clarified the discrepancies finding a significant relationship between renal scars and a PCT ≥0.5 ng/mL (aOR = 2.1; 95% CI, 1.4–3.9) when pooling all the data together [29]. High PCT offered a 79% (95% CI, 71–86) sensitivity and a 41% (95% CI, 34–48) specificity [29]. Before clinical use of PCT to identify children at high risk of renal scarring after UTI and selectively perform late DMSA-scan, validation studies and threshold analyses are needed to derive an evidence-based clinical decision rule.

## 5. PCT and VUR

PCT has been demonstrated to be correlated to both APN [28, 29] and late renal scars [29] on one hand, and on another hand VUR is thought to be related to a higher risk of APN and late renal scar [33]. PCT would thus be of interest to also predict VUR in children with UTI and could find a place in the investigation strategy. A first single-centre study demonstrated a significant relationship between both all-grade and high-grade VUR and a PCT ≥0.5 ng/mL (aOR = 4.9; 95% CI, 1.7–14.0—Table 3) [34]. High PCT sensitivities were 85% (95% CI, 70–94) and 92% (95% CI, 65–99) for all-grade and high-grade reflux, respectively, with 44% specificity (95% CI, 35–54) [34]. However, a strong controversy was raised regarding the urine collection techniques used (sterile bags), suggesting that this might have overestimated results [35]. These results were confirmed in two multicentre studies, adjusting the relationship between VUR and PCT on either urine collection techniques and early DMSA scan results [36, 37]. Complex relationships between UTI, renal parenchymal involvement, and VUR may happen, and one can argue that PCT may only reflect the presence of renal parenchymal involvement during UTI, and by the way identify children with VUR as VUR is known as a risk factor for acute pyelonephritis and renal scarring. However, interestingly, the second European validation we performed demonstrated that, in children with a proven acute pyelonephritis, PCT is significantly higher in children with high-grade VUR (≥3) than in those with low-grade or no VUR (1.5 ng/mL, interquartile range: 0.5–4.0 versus median 4.0 ng/mL, inter-quartile range: 1.5–7.0, resp.; *P* < .001). This definitively confirmed that PCT is a reasonable predictor for VUR. As recent publications demonstrated the absence of benefit for treating low-grade VUR, the target was then more focused on the early identification of children with only high-grade VUR. Finally, PCT was combined with renal ultrasonography findings, in order to take into account that clinicians believe in the high accuracy of ultrasonography, and a clinical decision rule was derived and internally validated [38]. Based on indirect relationships between VUR, scar and PCT, and statistical work, we ended up with a clinical tool combining PCT with renal ultrasonography that may be useful for clinicians to predict high-grade VUR in children with UTI and then selectively prescribe them a cystography. The clinical decision rule proposed in its current form that, for children aged 1 month to 4 years with a first febrile UTI, cystography should be performed in cases with ureteral dilation (i.e., ureter visible whatever its dilation) and a serum PCT level ≥0.17 ng/mL, or without ureteral dilatation (i.e., ureter not visible) when the serum PCT level ≥0.63 ng/mL. The rule had a sensitivity of 86% (95% CI, 74–93) with a specificity of 47% (95% CI, 42–51). The introduction of a renal ultrasonography criterion in the decision rule may be arguable, as the diagnostic accuracy of renal US for VUR is debated, with conflicting results in literature [39]. However, this decision was made based on the strong beliefs of pediatricians that renal US predicts well VUR in order to derive a potentially well-accepted rule, and the ultrasonographic criterion with the higher diagnostic accuracy for VUR was chosen [40]. However, it needs an external validation and a measure of its clinical impact, as recommended in the standards for building such tools [41, 42] before its daily and safe use in clinical practice.

## 6. Conclusion

PCT, a reliable marker of bacterial infections, would find a place in the complex and still debated picture of which examination should be performed after UTI in children. Indeed, PCT demonstrated a reasonable diagnostic accuracy for both APN and renal scarring and has been proposed as a key tool for a clinical decision rule to predict high-grade VUR. However, more research is warranted to understand more in depth the physiopathologic mechanisms of PCT in bacterial infection and especially UTI on one hand, and validations, threshold analyses, and impact studies are required before daily and safe use of PCT on another hand. Moreover, the exact place of this biomarker probably would need to be refined when pediatrics societies would agree on a diagram of the order and timing of investigations after UTI, but PCT may be certainly found playing a role.

## Figures and Tables

**Table 1:** Studies on diagnostic accuracy of procalcitonin for acute pyelonephritis in children with urinary tract infection.

Study	City, country	*n*	Delay for early DMSA scan	Threshold used for PCT (ng/mL)	Results
Benador, 1998 [22]	Geneva, Switz.	80	Within 5 days	>0.6	Se: 70%; Sp: 83%

Gervaix, 2001 [21]	Geneva, Switz.	57	Within 5 days	≥0.5	Se: 74% (56–87); Sp: 85% (62–97)

Smolkin, 2002 [24]	Afula, Israel	64	Within 7 days	≥0.5	Se: 94%; Sp: 90%; PPV: 86%; NPV: 98%

Pecile, 2004 [16]	Udine, Italy	100	Within 5 days	≥0.5	Se: 91%; Sp: 70%; PPV: 78%; NPV: 87%
				≥0.8	Se: 83%; Sp: 94%; PPV: 94%; NPV: 83%
				≥1.0	Se: 81%; Sp: 94%; PPV: 94%; NPV: 81%

Gurgoze, 2005 [18]	Firat, Turkey	76	Within 7 days	>0.5	Se: 58%; Sp: 76%

Bigot, 2005 [19]	Lille, France	42	Within 3 days	≥0.5	Se: 100%; Sp: 87 %; PPV: 86 %; NPV: 100%

Tuerlinckx, 2005 [20]	Yvoir, Belgium	63	Within 3 days	≥0.5	Se: 68%; Sp: 23%

Güven, 2006 [26]	Antalya, Turkey	33	Within 3 days	≥1.7	Se: 46%; Sp: 77%
				≥0.5	Se: 65%; Sp: 38%; PPV: 62%; NPV: 42%
				≥1.0	Se: 77%; Sp: 45%; PPV: 48%; NPV: 75%
				≥2.0	Se: 100%; Sp: 43%; PPV: 24%; NPV: 100%

Karavanaki, 2007 [27]	Athens, Greece	58	Within 7 days	≥0.5	Se: 94%; Sp: 76%; PPV: 68%; NPV: 96%
				≥0.8	Se: 94%; Sp: 88%; PPV: 80%; NPV: 96%
				≥1.0	Se: 94%; Sp: 100%; PPV: 100%; NPV: 97%

Belhadj-Tahar, 2008 [23]	Toulouse, France	183	Day 4		PCT were significantly higher in patients with early scintigrahic alteration (7.85 versus 2.36 ng/mL)

Kotoula, 2009 [31]	Thrase, Greece	57	Within 7 days	≥0.85	Se: 89%; Sp: 97%, PPV: 56%; NPV: 91%

Bressan, 2009 [17]	Padova, Italy	72	Within 7 days		Results focused on scars

Nikfar, 2009 [25]	Ahvaz, Iran	100	Within 7 days	≥0.5	Se: 77% (65–87); Sp: 89% (75–97);
					PPV: 92% (81–98); NPV: 71% (56–83)

DMSA scan, Tc-99m dimercaptosuccinic acid scan; NPV, negative predictive value; PCT, Procalcitonin; PPV, positive predictive value; Se, Sensitivity; Sp, Specificity.

**Table 2:** Studies on diagnostic accuracy of procalcitonin for late renal scars in children with urinary tract infection.

Study	City, country	*n*	Delay for early DMSA scan	Results
Benador, 1998 [22]	Geneva, Switz.	80	3 months	Mean PCT values of 1.6 ± 0.6 mg/L in the totally reversible DMSA lesions versus 7.7 ± 3.0 mg/L in the partially reversible group (*P* = .02).

Pecile, 2004 [16]	Udine, Italy	100	6 months	PCT levels for children with totally reversible lesions on followup scans (3.3 ± 3.5 ng/mL) versus those for children with renal scars (7.45 ± 8.4 ng/mL; *P* = .04).

Gürgöze, 2005 [18]	Firat, Turkey	76	6 months	Control DMSA scan performed showed that scar tissue developed in 4 cases (11%).

Tuerlinckx, 2005 [20]	Yvoir, Belgium	63	6 months	The median PCT level was not statistically different between children with totally and partially reversible lesion(s) (*P* = .3).

Güven, 2006 [26]	Antalya, Turkey	33	3–6 months	On 19 followup scans, 13 (68%) showed complete resolution. On the 6-month scans, five of 21 patients (24%) had renal scars. No correlation with PCT levels was studied.

Karavanaki, 2007 [27]	Athens, Greece	58	6 months	PCT values (3.08 mg/L versus 5.3 mg/L; *P* = .05) were significantly lower in the group with totally reversible renal lesions.

Kotoula, 2009 [31]	Thrase, Greece	57	6 months	The PCT level was significantly greater in the patients with persistent renal lesions (median PCT level of 10.4 ng/mL, range 1.6–13.0) than in those with total regression (1.9 ng/mL, range 0.7–10.0; *P* = .005).

Bressan, 2009 [17]	Padova, Italy	72	12 months	Patients with persistent lesions had significantly higher PCT values (2.3 ng/mL, IQR: 1.0–11.6) than those without permanent renal lesions (0.5 ng/mL, IQR: 0.2–1.4; *P* = .007).

DMSA, Tc-99m dimercaptosuccinic acid scan; IQR, Interquartile range; PCT, Procalcitonin.

**Table 3:** Studies on prediction accuracy of procalcitonin for vesicoureteral reflux in children with urinary tract infection.

Study	City, country	*n*	All-grade VUR			High-grade VUR		
			aOR*	Sensitivity	Specificity	OR	Sensitivity	Specificity
Prevur II study [34]	Paris, France	136	4.9 (1.7–14.0)	85% (70–94)	44% (35–54)	8.7 (1.2–382)	92% (65–99)	44% (35–54)
Prevur III study [36]	Multicentre	398	2.5 (1.4–4.4)	75% (66–83)	43% (37–48)	24.7 (1.5–415)^†^	100% (81–100)	43% (37–48)
Prevur IIIb study [37]	Multicentre	526	—	—	—	2.5 (1.1–5.4)*	83% (71–91)	43% (38–47)
Prevur V study** [38]	Multicentre	494	—	—	—	5.2 (2.4–11.3)	86% (74–93)	47% (42–51)

*aOR: Adjustment on usual cofactors of interest: age, gender, family history, renal ultrasonography results, and urine collection techniques (in Prevur III study [36]), or early DMSA scan results (in Prevur IIIb study [37]).

**This study built a rule combining PCT and ureteral dilation on renal ultrasonography and studied its prediction accuracy for high-grade VUR.

^†^Relationship between high PCT and grade 4 and 5 VUR.

All results were given with the 95% confidence interval in brackets.

Abbreviations: aOR, adjusted odds ratio; VUR, vesicoureteral reflux.

## Abbreviations

APN: Acute pyelonephritis
OR: Odd Ratio
PCT: Procalcitonin
UTI: Urinary tract infection
VUR: Vesicoureteral reflux.
